# Age and Ageism in the COVID-19 Pandemic: What Does the Data Show?

**DOI:** 10.1093/geroni/igab046.2294

**Published:** 2021-12-17

**Authors:** Usha Dhakal, Suzanne Kunkel

**Affiliations:** Miami University, Oxford, Ohio, United States

## Abstract

Gerontologists were quick to call out the resurgence of ageism that was reflected in the paternalistic, overgeneralized, and deficit views of aging that dominated discussions about age-associated risks of the disease and its consequences. One manifestation of the blunt and potentially ageism-promoting use of age in data about the virus is the failure to routinely distinguish the independent role of age alone, separate from its association with comorbidities. A related problem is the use of broad age categories, which can also mask the role of specific comorbidities. To address that gap, this study uses data from Centers for Disease Control and Prevention, as of Feb 21, 2021 to calculate age-specific COVID-19 death rates (ASDR) and compare the extent to which comorbid conditions potentially associated with COVID-19 deaths were listed on death certificates. Findings showed that the ASDR was significantly higher for those 85 years and over (2249.96 per 100,000); the rate was 802.66 for 75-84 and 312.78 per 100,000 for 65-74. Death certificate information revealed that influenza and pneumonia was the major contributing comorbidity to COVID-19 deaths across all three age groups; (listed on 49% of the death certificates for those 65-74 who died with COVID-19, 46% of those 75-84, and 38% of those 85 and over). Future studies should be more precise about the use of age/age groups, about the rationale for those designations, and about the impact of age separate from comorbidities. Broad use of an arbitrary age as a proxy for frailty and illness contributes to ageism.

